# Ossification of Cranial Epidural Hematomas: A Systematic Review of Management Strategies and Presentation of an Illustrative Case

**DOI:** 10.1089/neur.2024.0065

**Published:** 2024-08-22

**Authors:** Insa K. Janssen, Julien Haemmerli, Andrea Bartoli, Melvin Joory, Emily Richards, Karl Schaller, Aria Nouri

**Affiliations:** ^1^ Department of Neurosurgery, Hôpitaux Universitaires de Genève, Geneva, Switzerland.; ^2^ Department of Radiodiagnostics and Interventional Radiology, CHUV, Lausanne, Switzerland.; ^3^ School of Clinical Medicine, University of Cambridge, Cambridge, United Kingdom.

**Keywords:** calcification, chronic epidural hematoma, cranial trauma, ossification

## Abstract

The presence of a calcified or ossified chronic cranial epidural hematoma (EDH) is rare and has been described in only a few case reports in the literature. Consequently, clear treatment strategies remain elusive and may entail conservative and surgical approaches. In this study, we performed a systematic review of reported cases to evaluate the clinical course and treatment options for these patients. A comprehensive systematic search of two databases was performed, and information on patient characteristics, symptomatology, and treatment was extracted from eligible articles. A total of 56 cases were included in our analyses. Forty patients were male, 16 were female, with an average age of 21.38 years at the time of diagnosis. Assumed etiology was previous trauma in 35 cases, previous cranial surgery in 17 patients, and birth trauma and epidural bleeding after the utilization of the Mayfield clamp in 1 case each. The origin remained unclear in two cases. The time between trauma or surgery and diagnostics ranged between one and a half weeks and 50 years, with a median of 4 years (SD 9.8 years). The symptoms were very heterogeneous, ranging from acute neurological deterioration to chronic symptoms. In 15 cases, patients were asymptomatic, and cranial imaging was performed as part of a new trauma or a screening for other disease. Forty-one patients received surgical treatment by craniotomy and hematoma evacuation, and 13 patients were treated conservatively. In two cases, the liquid hematoma portion was aspirated through a burr hole. The localization of calcified or ossified EDH was mainly supratentorial. Young male patients most commonly present with calcified or ossified EDH after trauma, according to the epidemiological trend of acute EDH. Clinical presentation varies from asymptomatic to severe neurological deficits and signs of increased intracranial pressure. There is no standardized treatment; decisions must be made on an individual basis.

## Introduction

Calcified or ossified cranial epidural hematomas (OCEHs) are rare and have been described in only a few case reports in the literature, typically in children.^1,2^ Intuitively, this ossification is believed to represent an evolution of chronic and untreated epidural hematomas (EDHs). However, it is unclear if OCEH occur due to the progression of smaller hematomas managed conservatively, undiagnosed hematomas, or other causes. Given their rare occurrence, there are no specific management guidelines, and treatment strategies may range from conservative treatment to surgical intervention.^2^

Acute epidural hematomas (aEDHs) occur in 2% of all head injuries and up to 15% of all severe cranial trauma in adults.^3^ In the pediatric population, aEDH represents 2–3% of all head injuries, with a mean age between 6 and 10 years, and it is rare among infants under the age of 12 months.^4,5^ Mortality has been reported to be around 5% in children and between 7 and 12.5% in adults.^6^ The origin of bleeding is typically due to disruption of the middle meningeal artery and its branches or venous rupture and, therefore, can result in rapid neurological decline (unconsciousness) due to a mass effect, with possible transient periods of neurological recovery (consciousness) due to cerebral perfusion pressure adaptation, referred to as lucid intervals.^7^ Due to anatomical characteristics, particularly tight adherence of the dura mater to the inner table of the skull, aEDHs are considered to be less common in children or elderly.^8^

Treatment of aEDH can range from conservative management to neurosurgical emergencies, depending on the clinical presentation. In adults, general guidelines recommend surgical treatment in cases of a hematoma volume greater than 30 ml, regardless of the Glasgow Coma Scale (GCS) score or GCS <9, with pupillary abnormalities such as anisocoria independent from volume. Asymptomatic patients can be observed but require surveillance with or without control imaging.^9^ However, in children, the clinical course is more gradual, and an associated skull fracture is rare.^4^ Furthermore, due to a more nonspecific clinical presentation, they can present a diagnostic challenge and may remain undiagnosed in some cases.^4^

The precise mechanism of the osseus transformation of hematoma remains unclear.^10^ It is known that damage to vascularized tissue like bone or dura initiates a tissue response including inflammation, repair, and remodeling.^10^ This process is assumed to be more rapid in children,^10^ depending on the type and side of the injury, patient’s age, and their metabolic status.^10^ Most of the growth of the skull takes place until the age of 7 and then continues in a linear fashion until adulthood, and diploe appears by 4 years and reaches maximum at 35 years.^10^ It is assumed that calcification/ossification may start with the formation of fibroblast layers adherent to the dura as early as 4 days after bleeding.^6^ This then develops into a connective tissue layer, which undergoes hyalinization and subsequent calcium deposition under conditions of poor perfusion or malabsorption of the hematoma content.^11,12^ Some authors describe the start of ossification between the junction of the hematoma and the capsule.^10,13,14^ Association with metabolic bone disease and endocrinological or coagulation disorders has not been described.^11^

Characteristic presentation in a skull radiograph or scan is a double-outlined contour representing bone formation and calcification of the hematoma capsule adjacent to the dura.^10^

This ossification is not considered to be unique to untreated EDHs. Ossification of cephalohematomas, which are a collection of blood between the skull and the pericranium (often caused by instrument assisted birth trauma),^15,16^ and subdural hematomas (generally in children or young adults, and typically after shunt surgery or nonaccidental head injury) have also been reported.^17^ Ossification of cephalohematoma has been reported to occur in 3–5% of cases,^17^ whereas ossification of subdural hematomas has been reported in up to 0.3–2.7% of cases.^18^ There is some controversy regarding the optimal treatment strategy for calcification or ossification of chronic subdural hematoma, and surgery is recommended in cases of neurological symptoms.^19^ Calcified cephalohematoma may cause significant distortion of the calvarium requiring surgical correction, which is the treatment of choice in these cases.^17,19^

Given their rarity and the incomplete understanding of their natural history and management strategies, this study aims to conduct a systematic review of the literature to (1) synthesize reports of OCEH cases and (2) evaluate the clinical course and treatment options for these patients.

To illustrate the clinical relevance of the topic, we also present a rare case of OCEH.

## Case

**FIG. 1.**
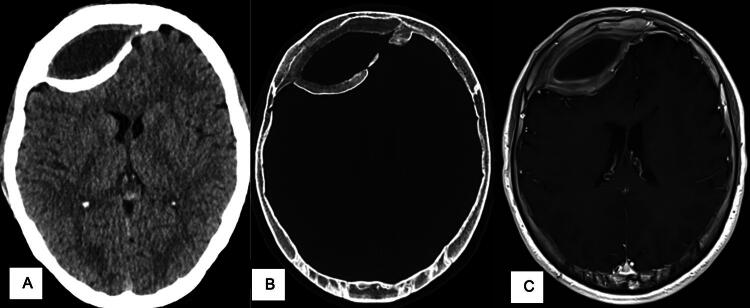
**(A, B)** First cranial scan in 2020 showing a biconvex frontal right ossification, compatible with an ancient calcified epidural hematoma. **(C)** A subsequent magnetic resonance tomography shows a compressive effect of the hematoma on the right frontal lobe.

A 22-year-old male, known for chronic alcohol abuse and the notion of osteogenesis imperfecta, presented to the hospital in 2020 after assault-related head trauma and loss of consciousness. The neurological exam was unremarkable. The patient has a history of multiple episodes of cranial trauma, the most recent of which occurred 3 years earlier. A cranial computer tomography (cCT) at the time did not show any intracranial hemorrhage or fracture. A new cCT undertaken at the emergency department shows a biconvex frontal right ossification (Fig. 1.A,B), compatible with a calcified EDH. A subsequent cranial magnetic resonance tomography shows a compressive effect of the calcified hematoma on the right frontal lobe. Evaluation based on neuropsychological examination at follow-up showed mild-to-moderate problems according to the criteria of the Association of Swiss Neuropsychologists. An indication for surgery without urgency was made due to the compressive effect, but the patient did not attend the follow-up for planning the surgery despite multiple reminders.

**FIG. 2.**
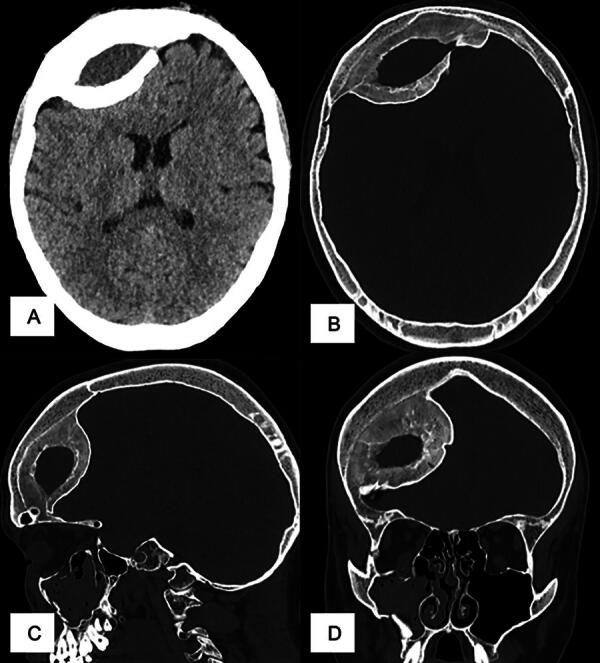
Cranial scan in 2023 after a new cranial trauma: persistence of the right frontal ossified epidural hematoma in the form of a biconvex lens. The thickness of calcification has increased, whereas the overall volume and mass effect remain the same (**A**: axial soft tissue window, **B**: axial bone window, **C**: sagittal bone window, coronal bone window).

However, he presented to the emergency room due to a new head trauma 3 years after the diagnosis of the calcified hematoma. He did not present new symptoms except for some headaches due to the new trauma. Cranial CT showed the persistence of the calcified hematoma. The overall volume and mass effect were the same, with the thickness of the calcification increasing while the liquid content decreased (Fig. 2. A–D).

## Materials and Methods

### Eligibility criteria

Table 1 provides a summary of the eligibility criteria used in this review. We extracted data to answer the following and key questions.

**Table 1.** Inclusion and Exclusion Criteria

	Inclusion	Exclusion
Patients	•Patients diagnosed with calcified or ossified epidural hematoma treated surgically or conservatively	•Patients diagnosed with acute epidural hematoma, calcified or ossified subdural hematoma/galeahematoma
Study design	•Case reports •Case series •Non-English language articles	•Review articles •Opinions •Animal studies

Q1. *What kind of patients are susceptible to developing a calcified/ossified epidural cranial hematoma (age, comorbidities, bone, or endocrinological diseases)?*

Q2. *Which kind of symptoms are the most common? (Is there a volume threshold before symptoms?)*

Q3. *What is the natural history and pathophysiology?*

Q4. *What treatment/management is recommended?*

### Information sources

The databases searched were Ovid MEDLINE(R) (1946 to December Week 3 2020, search was run 30.12.2020) and PubMed (search was run 30.12.2020).

As the search was already carried out in 2020, we carried out a new search for the years 2021–2023 before publication of the results (search was run 01.12.2023).

### Search

An experienced medical librarian performed a comprehensive search of two databases after consultation with the lead authors and a Medical Subject Heading analysis of key articles provided by the research team. In each database, we used an integrative process to translate and refine the searches. Both English and foreign language articles were eligible.

The formal search strategies used relevant controlled vocabulary terms and synonymous-free text words and phrases to capture the concepts of ossified, calcified intracranial EDH. The full strategy for OVID MEDLINE and PubMed is available in Supplementary Appendix S1.

### Study selection

**FIG. 3.**
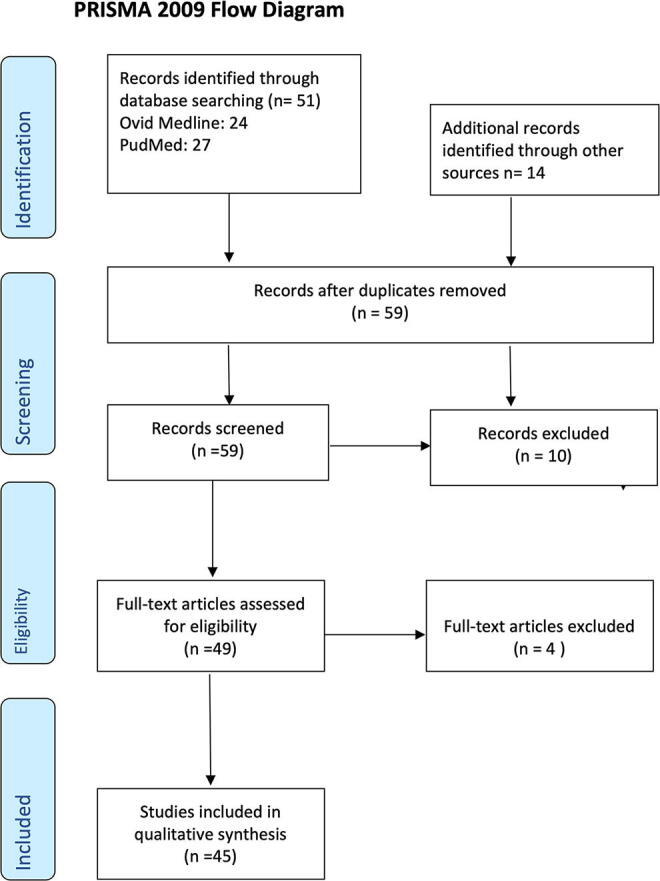
PRISMA flow diagram. Overview of search strategy carried out in 2020 (search was run 30.12.2020).

Two separate screeners (I.J. and M.J.) evaluated the titles and abstracts of the eligible articles in a standardized manner. The abstracts were classified as relevant, possibly relevant, or not relevant according to the inclusion criteria (Table 1). A full-text investigation of the possibly relevant studies was done for further clarification, and disagreement between reviewers was resolved through discussion. Search strategies are available from the authors, and the flowchart per PRISMA (Preferred Reporting Items for Systematic Reviews and Meta-Analyses) is presented in Figure 3. The included articles are shown in Table 2.

**Table 2.** Included Articles

	Author(s), year of publication	Title	Language	Type of article	Journal
1	Kotil & Akçetin, 2006^10^	Asymptomatic chronic ossified epidural hematoma in a child: a rare entity.	*English*	Case report (1 case)	Turkish journal of trauma and emergency surgery
2	Akhaddar & Boulahroud, 2015^20^	Ossified chronic epidural hematoma of the posterior fossa	*English*	Case report (1 case)	Pan African Medical Journal
3	Kumar & Mittal, 2014^21^	Post-traumatic chronic ossified extradural hematoma: a rare case report.	*English*	Case report (1 case)	Romanian Neurosurgery
4	Miyazaki & Akagwa, 1968^22^	Calcified Epidural Hematoma	*English*	Case report (1 case)	Neurologia medico-chirurgica
5	Bishnoi et al., 2018^23^	Ipsilateral two spontaneous chronic calcified epidural hematoma	*English*	Case report (1 case)	Asian Journal of Neurosurgery
6	Cambria et al., 1985^24^	Ossified epidural hematoma, report of a case with epilepsy	*English*	Case report (1 case)	J Neurosurg Sci
7	Chang et al., 2002^15^	Chronic epidural hematoma with rapid ossification	*English*	Case report (1 case)	Childs Nervous System
8	Chen et al., 2016^18^	Bilateral large chronic ossified epidural hematoma after ventriculoperitoneal shunt: A special case report and treatment management	*English*	Case report (1 case)	International Journal of Clinical and Experimental Medicine
9	Claiborne et al., 2015^2^	Extradural Ossification Following Epidural Hematoma in Children: A Rare But Significant Entity	*English*	Case report (1 case)	The journal of craniofacial surgery
10	Datta & Sharma, 2016^25^	Calcified Epidural Hematoma in an Adult Patient: A Case Report	*English*	Case report (1 case)	Indian Journal of Neurotrauma
11	Dawar et al., 2013^26^	Same side double chronic calcified epidural hematoma: Case report and review of literature	*English*	Case report (1 case)	Neuroly India
12	De Oliveira et al., 2008^8^	Large chronic epidural hematoma with calcification: a case report	*English*	Case report (1 case)	J. Trauma/ Journal of Trauma-Injury Infection & Critical Care
13	Djoubairou et al., 2018^27^	Chronic calcified extradural and subdural hematoma following a ventriculoperitoneal shunt placement	*English*	Case report (1 case)	Neurology India
14	Agrawal & Giri, 2018^28^	Dual Chronic Ossified Epidural Hematomas Presented with Seizures 23 Years after Head Injury in an Adult Male: Case Report and Literature Review	*English*	Case report (1 case)	Indian Journal of Neurotrauma
15	Han, 2015^29^	Conservative management of a rapidly calcifying epidural hematoma in a young male patient	*English*	Case report (1 case)	Interdisciplinary Neurosurgery: Advanced Techniques and Case Management
16	Iwakuma & Brunngraber, 1974^14^	Extradural ossification following an extradural hematoma: Case report	*English*	Case report (1 case)	JNS
17	Kawata et al., 1994^30^	Ossified epidural hematomas: report of two cases	*Japanese*	Case report (2 cases)	Neurological Surgery
18	Kia-Noury & Wiedenmann, 1963^31^	Intrakranielle Verkalkungen bei epiduralen Hämatomen	*German*	Case report (1 case)	Neurochirurgia
19	Kim et al., 2014^32^	Rapid ossification of epidural hematoma in a child: a case report	*English*	Case report (1 case)	Korean J Neurotrauma
20	Leclercq & Rozycki, 1979^33^	Chronic calcified epidural hematoma in a child	*English*	Case report (1 case)	R I Med J/ Rhode Island medical journal
21	Lee et al., 2014^1^	Serial CT findings of a rapidly calcified epidural hematoma in a young adult: a case report	*English*	Case report (1 case)	J Neuroimaging
22	Mathuriya et al., 1989^34^	Ossified epidural hematoma. Report of two cases	*English*	*Case report (2 cases)*	Clinical Neurology & Neurosurgery
23	Matsumoto et al., 1985^35^	Chronic Epidural Hematoma: Report of Two Cases	*Japanese*	*Case report (2 cases,* 1 edh calcified)	Neurologia medico-chirurgica
24	Mishra et al., 2014^36^	Shunt site chronic calcified extradural hematoma: An avoidable complication	*English*	Case report (1 case)	Journal of Pediatric Neurosciences
25	Nagane et al., 1994^13^	Ossified and calcified epidural hematoma incidentally found 40 years after head injury: case report	*English*	Case report (1 case)	Surgical Neurology
26	Nitta et al., 1984^37^	A case of calcified epidural hematoma	*Japanese*	Case report (1 case)	Japanese Journal of Clinical Radiology
27	Parkinson et al., 1980^38^	Ossified epidural hematoma: Case report	*English*	Case report (1 case)	Neurosurgery
28	Roganovic et al., 1992^39^	Ossified chronic epidural hematoma	*Serbian*	Case report (1 case)	Military-medical and pharmaceutical review
29	Sakai, 1977^40^	Calcified epidural hematoma—report of a case incidentally found 16 years after head injury	*Japanese*	Case report (1 case)	Neurological Surgery
30	Sakurai et al., 1998^41^	A case of ossified epidural hematoma	*Japanese*	Case report (1 case)	Japanese Journal of Neurosurgery
31	Schumacher, 1982^42^	Ungewöhnliche radiologische und klinische Befunde bei verkalkten epiduralen Hämatomen/ Unusual radiological and clinical findings in a calcified chronic extradural hematoma	*German*	*Case series (3 cases)*	Neurochirurgia
32	Erdogan et al., 2003^43^	Rapidly calcifying and ossifying epidural hematoma	*English*	Case report (1 case)	Pediatric Neurosurgery
33	Seyıthanoglu, 2010^44^	Chronic ossified epidural hematoma after ventriculoperitoneal shunt insertion: A case report	*English*	Case report (1 case)	Turkish Neurosurgery
34	Siedschlag & Schulz, 1982^45^	Über ein verkalkendes chronisches epidurales Hämatom	*German*	Case report (1 case)	Zentralblatt fur Neurochirurgie
35	Trivedi & Hiran, 2010^46^	Calcified epidural hematoma in pediatric age-group: A report of two cases	*English*	Case report (2 cases)	Journal of Neurosciences in Rural Practice-
36	Trodi et al., 2007^47^	Chronic calcified extradural hematoma	*French*	Case report (1 case)	J Neuroradiol
37	Wishler et al., 1964^48^	Ossified epidural hematoma following posterior fossa exploration: report of a case	*English*	Case report (1 case)	JNS
38	Yeh et al., 2014^49^	Ossified Epidural Hematoma	*English*	Case report (1 case)	Journal of Emergency Medicine/ VISUAL DIAGNOSIS IN EMERGENCY MEDICINE
39	Yoshida et al., 1985^50^	A case with postoperative calcified epidural hematoma	*Japanese*	Case report (1 case)	Neurological Surgery
40	Yu et al., 2008^16^	Rapidly calcified epidural hematoma in a neonate	*English*	Case report (1 case)	Journal of Korean Neurosurgical Society
41	Zhang et al., 2015^51^	Gigantic ossified chronic epidural hematoma and contralateral postoperative subdural hematoma: a case report and literature review	*English*	Case report (1 case)	British Journal of Neurosurgery
42	Choudhary et al., 2012^52^	Epidural Hematoma with Ossification: Two Case Reports	*English*	Case report (2 cases)	Pakistan Journal of Radiology
43	Jain et al., 2012^53^	Chronic ossified extradural hematoma on the opposite side of the ventriculoperitoneal shunt procedure: A rare case report	*English*	Case report (1 case)	Saudi Journal for Health sciences
44	Mehra et al., 2017^54^	Calcified Extradural Hematoma with overlying Subcutaneous Swelling: A Rare Case Report	*English*	Case report (1 case)	IOSR Journal of Dental and Medical Sciences (IOSR-JDMS)
45	Bayri et al., 2009^12^	Iatrogenic chronic calcified/ossified epidural hematoma: case report	*English*	Case report (1 case)	J Nervous Sys Surgery
46	Sinha & Borkar, 2008^6^	Chronic calcified extradural hematoma in a child: Case report and review of literature	*English*	Case report (1 case)	Indian Journal of Neurotrauma (IJNT)
47	Sheyin et al., 2021^55^	Unusual presentation of epidural haematoma with ossified border on computed tomography scan.	*English*	Case report (1 case)	Nigerian Journal of Basic and Clinical Sciences
48	Banga et al., 2021^56^	Chronic Epidural Hematoma: Still a Rare Entity?	*English*	Case series (1 case of edh with ossification)	Indian Journal of Neurotrauma
49	Kanikomo et al., 2023^57^	Intracranial Calcified Extradural Hematoma about a Case.	*English*	Case report (1 case)	Open Journal of Modern Neurosurgery

### Data extraction and synthesis

The following data were extracted by the primary author of this study: patient sample and characteristics, assumed etiology (previous trauma or surgery), time between trauma/surgery and first diagnostic of calcification, symptomatology, comorbidities, treatment of OCEH, in case of surgical treatment timing of surgery, imaging, localization, volume, and histological findings.

## Results

### Study selection

Our systematic search identified 51 unique articles. Fourteen additional records were identified through other sources. After the removal of duplicates, 59 abstracts were screened. After reviewing the titles and abstracts, 10 articles did not meet our inclusion criteria. Following the full-text investigation, additional four articles were excluded because they did not provide case descriptions. A total of 45 articles were deemed relevant following the review process.

As the search was already carried out in 2020, we carried out a new search for the years 2021–2023 (search was run 01.12.2023), which resulted in four new articles.

### Study characteristics

All studies were case reports or case series. The included articles are summarized in Table 2. There were no randomized controlled trials or prospective studies, 38 articles were in English, 6 in Japanese, 3 in German, and 1 each in French and Serbian. The year of publication varies from 1963 to 2023, and foreign language articles were translated using Google translator.

### Results of individual studies

Forty-nine articles, reporting 55 cases of calcified or OCEH, were included in the screening process.

By adding our clinical case, a total of 56 cases were included in our analyses. Data extraction from the articles is detailed in Table 3. The majority of patients were male (40 vs. 16 female). The average age was 21.38 years (SD 15.07, range 26 days−73 years) at the time of diagnosis. The youngest female patient was 26 days old. Details of the patients’ history are summarized in the supplemental data.

**Table 3.** Localization of Hematomas

Localization	
Left frontal	10
Bifrontal	4
Posterior fossa, left cerebellar	1
Right hemisphere	1
Left occipital and suboccipital	1
Right parietal	9
Right frontal	6
Left parietal	11
Left frontoparietal	1
Left temporal	1
Right temporal	1
Left parietooccipital	1
Parietooccipital right	1
Left frontotemporal	2
Left temporoparietal	1
2 localizations: left parietal and left frontal	1
2 localizations: right frontal and left parietal	1
2 localizations: right frontal and parietal	1
2 localizations: right frontal and left parietal	1
2 localization: frontal right, parietal left	1

#### Q1. What kind of patients are susceptible to developing a calcified/ossified epidural cranial hematoma (age, comorbidities, bone, or endocrinological diseases)?

No bone or endocrinological, metabolic, or other comorbidities were found to be predictive. Most patients were confirmed to be in good health without abnormalities in the routine biological examination (*n* = 37), whereas, only in two cases, extended studies to assess a coagulation disorder (protein S and C deficiency, factor V Leiden, and antithrombin III) were performed and described as normal. In five cases, no biological examination was mentioned, and it remains unclear if routine biological or special examinations were obtained. Individual comorbidities were mentioned in some cases, but, due to the small number of cases, no correlations could be established. In one case each, an endocrinological disorder (pubertas praecox) and a coagulation disorder (thrombocytopenia purpura) were reported in childhood. In one case, there was a suspicion of osteogenesis imperfecta and confirmed alcohol abuse. Among the adult patients, alcohol abuse was mentioned in two other cases, which is a potential risk factor for coagulation problems.

In most cases (*n* = 35), the cause of the epidural hematoma was a previous cranial trauma. 17 patients had a history of cranial surgery (due to tumor *n* = 7, hydrocephalus *n* = 8 or trauma *n* = 2). Birth trauma and intracranial vascular injury after application of the Mayfield clamp were the cause in one case each. In two cases the origin remained unclear.

The time between trauma or surgery and diagnosis ranged between one and a half weeks and 50 years, with a mean of 4 years (SD 9.8 years).

The localization of the hematoma was supratentorial in most cases (*n* = 55). Only in one case, the hematoma was left cerebellar; in five cases, there were two localizations (Table 3).

#### Q2. Which kind of symptoms are the most common? (Is there a volume threshold before symptoms develop?)

Symptoms at the time of diagnosis were very heterogeneous, ranging from acute neurological deterioration to chronic symptoms like developmental delay (Table 4). In 15 cases, patients were asymptomatic, and cranial imaging was performed because of a new trauma or a screening for other disease. Symptoms that led to imaging and thus to the diagnosis were seizures (*n* = 11), mild symptoms of increased intracranial pressure without neurological deficit (*n* = 10), mild-to-moderate symptoms of increased intracranial pressure with neurological deficit (*n* = 8), and cerebellar syndrome (*n* = 3). Two patients presented with rapid deterioration, with unilateral mydriasis in one case. In three cases, the calvaria deformity was recognized. In three cases, patients suffered from chronic or acute psychomotor abnormalities and mental and personality changings, whereas one of them showed learning difficulty since childhood. One patient was symptomatic from an acute or chronic calcified EDH due to a bike accident fall 2 days prior. Diagnostic imaging subsequently showed a calcified hematoma.

**Table 4.** Symptoms Leading to Diagnostics of Calcified Epidural Hematoma (EDH)

Symptoms leading to diagnostic of calcified EDH	
Asymptomatic	15
Mild symptoms of increased intracranial pressure (mild/moderate headache, with or without vomiting) without neurological deficit	10
Mild–to-moderate symptoms of increased intracranial pressure (mild/moderate headache, with or without vomiting) with neurological deficit	8
Cerebellar syndrome	3
Epileptic seizures	11
Calvarial deformity	3
Altered consciousness, deterioration with or without mydriasis unilateral within hours	2
Chronic psychomotor abnormalities, learning difficulty since childhood	1
Mental disorder, confusion, psychomotor abnormalities	1
Memory problems since 6 weeks+ some headache, personality changes	1
Headache and vomiting following fall from bike 2 days ago, hematoma with calcification asymptomatic (age unclear), diagnostic due to head trauma, symptoms due to acute component	1

A neuropsychological exam was conducted in only one case and showed mild-to-moderate problems. The patient refused surgical treatment, which is why no follow-up neuropsychological exam was performed. Electroencephalography (EEG) was performed in five cases, was not pathological in three cases, and presented an epileptic focus in two cases in the localization hematoma.

Information regarding hematoma volume was available in only 11 cases, ranging from 13.5 to 280 cm^3^. The thickness of ossification was provided in eight cases and ranged from 1 to 12 mm. Due to the limited data, no volume threshold could be identified.

#### Q3. What is the natural history and pathophysiology?

The time between initial trauma or surgery and diagnosis ranged between one and a half weeks and 50 years, with a mean 4 years (SD 9.8 years). The exact time of calcification cannot be determined, as there was a lack of structured follow-up or imaging in most of the reported cases.

Out of 35 patients who had suffered from a cranial trauma, only 21 had imaging after the initial accident the day of admission or a few days later. Out of these 21 patients, 5 received only an X-ray of the skull, with subsequent skull fractures diagnosed in three cases; in two cases, the X-ray was without pathological findings. Fourteen patients received a cCT, which revealed a skull fracture without bleeding in 1 case and an acute EDH in 11 cases. Two of them were operated on immediately. The other nine patients received follow-up by scan or clinical surveillance. The first signs of ossification were visible after 12 days in one case. Four of these nine patients were treated conservatively, and five developed symptoms related to the OCEH needing surgery. Radiological surveillance showed a reduction of the liquid portion of the OCEH in some cases, whereas others showed persistent or increasing ossification. In one case, the OCEH was fully absorbed, with the hematoma having merged into the inner table of the skull after 6 months. Follow-up for cases treated conservatively was not longer than 12 months.

Intraoperative findings were described in 24 cases. In most of these cases, the hematoma capsule was described as a hard layer adhered to the dura mater and without clear demarcation to calvarium. Nevertheless, in some cases, a good plane between dura and wall of hematoma could be found, and blunt dissection could be performed; in other cases, the dura was calcified and had to be removed at least partly with the hematoma. In one case, the mass was described to be fused with the skull. In another case, no capsule could be macroscopically identified. In some cases, hyperplasia of the skull itself was described. Calcification and ossification were described, especially on the side of the dura, as a newly formed bone at the inner border of the hematoma capsule covering the underlying dura. The inner part of the hematoma was a yellowish brown to dark brown liquified hematoma gelatinous mass, only partially liquified inside.

Histological examination was available in 17 cases, all providing similar findings. The hematoma showed connective tissue with inflammatory cells in the outer membrane and ossification with lamellar bone layers in the inner membrane. Osteoblasts and osteocytes were found in the calcified part on the side of the dura, in association with neovascularization. Areas of ossification, calcification, and fibro-collagenous areas were seen in the hematoma. Some areas showed normal and new bone in the inner shell, with bony lamellas in parallel to the calvarium, and partly normal hematopoietic marrow. A lamellar bone layer was continually formed between the dura and the calcified inner capsule. The ossified shell fused with the outer shell, consisting of the normal calvarium at the margin. Ossification was mainly seen on the side of the dura. Macrophages were present in the hematoma containing hemosiderin. The organized hematoma mass was composed of collagen tissue with calcification.

#### Q4. What treatment/management is recommended?

After diagnosis of OCEH, 41 patients received surgical treatment by craniotomy and hematoma evacuation, and 11 patients were treated conservatively. In two cases, only the liquid hematoma was drained by burr hole trepanation. In two cases, emergency surgery was performed due to progressive neurological symptoms. In the other cases, surgery was performed during the hospital stay, within a few days after admission. In two cases, surgery was indicated but refused by the patient and the patient’s parents. Three postoperative complications were reported and managed successfully: (1) intracerebral hematoma (revision surgery) and hydrocephalus (VP-shunt) in one patient, (2) shunt infection and ventriculitis, and (3) occurrence of a chronic subdural hematoma contralateral 2 months later, which had to be drained. In one case, neurological symptoms ceased, but seizures stayed and needed long-term medical treatment.

## Discussion

Calcified or OCEHs are rare, and the incidence remains unknown.^1,2^ In our literature search, we found 55 cases over a period of 60 years, published between 1963 and 2023. By adding our clinical case, a total of 56 patients were included in our analyses. The patients’ medical histories and symptoms at the time of diagnosis were very heterogeneous, which is partly due to the long period of time during which the reported patients were treated. Similar to the incidence of aEDH, which typically affects males in their 20s to 30s who have suffered head trauma, the majority of patients in our series were males with an average age of 21.38 years (SD 15.07, range 26 days−73 years).^3^ This is intuitive, as ossification is thought to be the consequence of unrecognized or progression of aEDH bleeding following cranial trauma. Indeed, this is largely supported by the fact that previous trauma was the predominant etiology reported in the reviewed cases (*n* = 35). However, what is not clear is when ossification occurs and when or if they will ever be symptomatic, especially since diagnosis in the series spanned weeks to decades to be discovered. Furthermore, symptoms can be quite variable and nonspecific. The delay of diagnosis or symptom development can be attributable to many factors: (1) potential variability in ossification progression over time between individuals, (2) symptoms may develop after additional episodes of trauma (due to decreased compliance and inability to absorb kinetic forces in the setting of reduced intracranial space), (3) incomplete hematoma resorption at last follow-up, and (4) no imaging was performed in the absence of a fracture in the skull X-ray (older reported cases) and was considered sufficient to rule out an intracranial hemorrhage. Recognizing that classical skull fractures are only present in very few cases of aEDH and that CTs are readily accessible and are undertaken even after minor trauma^2^ (with somewhat higher thresholds in the pediatric population to avoid radiation exposure), aEDHs are rarely missed nowadays.^56,58^

The nonoperated aEDH usually progresses to absorption in 3–15 weeks.^57^ The mechanism of resorption of extradural hematoma described in the literature ranges from the evolution of a fibrovascular membrane covering the dural face of the chronic EDH^59^ to arteriovenous shunts that develop in the extradural space during hemorrhage.^57^ D’Andrea et al. describe increasing interest in the conservative treatment of EDH in children and believe that the incidence of OECH may increase due to this in the coming years in the pediatric population.^58^

Despite existing theories, the mechanism of calcification/ossification is still unclear, and it is unknown why a small percentage of hematomas do not disappear and become calcified or ossified.^41^ No predisposing characteristics or comorbidities for the calcification of EDHs were found in this series of cases. From this series, it does appear, however, that the initiation of the ossification appears at the junction of the hematoma and capsule and is associated with local inflammation that results in the proliferation of fibroblasts.

A possibility of a similar entity to OCEH is the ossification/calcification of subdural hematomas. Although these are better known, they are also a rare entity, and their optimal management has also not yet been determined.^60^ It is known from these cases, however, that due to the “armored brain” phenomenon, there may be limited re-expansion of the brain after surgery,^60,61^ with some authors reporting no improvement after surgery after long-standing symptoms. It has thus been recommended that surgery should only be pursued when acute or progressive neurological symptoms occur,^60,62^ whereas others propose removing the lesion because of the potential risk of increasing irritation of the brain by thickening lamellar bone.^60^

With regard to OCEH, most of the reported cases reported in this review (73%, 41/56) were treated with varying surgical techniques. However, it is unclear if this rate of surgery is affected by the fact that these cases probably represented more severe forms and potential publication bias. Generally speaking, surgery is clearly indicated in patients with neurological symptoms due to the mass effect. However, decision-making is more difficult in cases of chronic symptoms and slight/minor psychomotor abnormalities, where reversibility is uncertain and the benefit of surgery is unclear. But some authors suggest surgical treatment even in the absence of mass effect or in asymptomatic patients due to a supposed higher risk of calcification/ossification progression.^57^ When considering surgery, these perceived benefits must be balanced with the possible surgical complications, including a potential lack of improvement in symptoms postoperatively, as well as brain contusion, bleeding, or the appearance of new neurological deficits when dissecting the calcified membrane tissue adherent to the cortical surface of the parenchyma.^60^

### Clinical management strategy

**FIG. 4.**
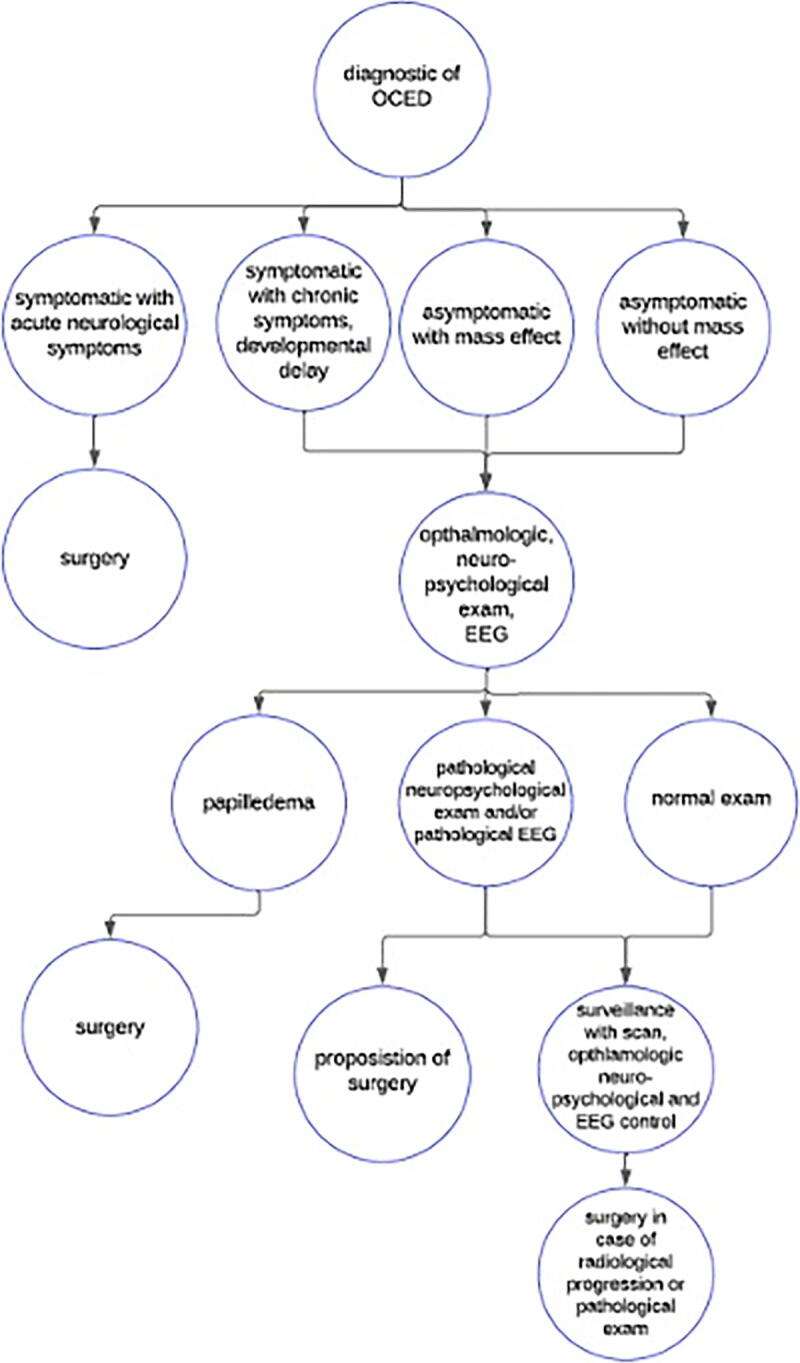
Proposed strategy for the management of patients with calcified or ossified cranial epidural hematoma.

The management of these cases must be carefully discussed, particularly in pediatric cases. Given that the symptoms of calcifications are unlikely to be rapidly transformative and place the patient at immediate risk, it is reasonable to follow these patients initially and intervene in the event of progression. Given the lack of literature on this subject, a structured guide and strategy on how to conduct follow-up and manage these patients remain unclear. For this reason, a proposed strategy for the management of these cases has been proposed here (Fig. 4).

It is proposed that surgery should be offered to patients with neurological symptoms and clear signs of progression. For patients, in whom OCEH is discovered incidentally or in the workup of minor symptoms, follow-up may be reasonable. Initial evaluation after the diagnosis as well as subsequent follow-up should include imaging, neuropsychological examination, EEG, and potential and clinical examination (which may include additional visual examination).

## Conclusion

OCEH after trauma or cranial surgery most commonly occurs in young male patients. Clinical presentation varies from asymptomatic, to mild and chronic, to severe acute neurological deficits with signs of increased intracranial pressure. There is no standardized treatment, and a practical treatment approach for their treatment has been proposed. Ultimately, treatment decisions should be based on a combination of examinations, and treatment recommendations should be tailored to the patient’s presentation. Unfortunately, this review was not able to determine specific factors that can identify which hematomas will ossify or calcify, and further work looking at the underlying pathophysiological and genetic factors would potentially be helpful but challenging to undertake given the rarity of this clinical entity.

## Abbreviations Used

aEDH: acute epidural hematoma
cCT: cranial computer tomography
cEDH: chronic epidural hematoma
EDH: epidural hematoma
EEG: electroencephalography
GCS: Glasgow Coma Scale
OCEH: calcified or ossified cranial epidural hematoma

## References

[1] Lee BH, Hwang YJ, Choi CY. Serial CT findings of a rapidly calcified epidural hematoma in a young adult: A case report. J Neuroimaging 2014;24(5):531–532. 24251759 10.1111/jon.12060

[2] Claiborne JR, Hoge MK, Wood BC, et al. Extradural Ossification Following Epidural Hematoma in Children: A Rare But Significant Entity. J Craniofac Surg 2015;26(5):1500–1503. 26106995 10.1097/SCS.0000000000001806

[3] Khairat A, Waseem M. Epidural Hematoma. In: StatPearls. Treasure Island (FL); 2022. 30085524

[4] Chowdhury SN, Islam KM, Mahmood E, et al. Extradural haematoma in children: Surgical experiences and prospective analysis of 170 cases. Turk Neurosurg 2012;22(1):39–43; doi: 10.5137/1019-5149.JTN.4550-11.1 22274969

[5] Jamous MA, Abdel Aziz H, Al Kaisy F, et al. Conservative management of acute epidural hematoma in a pediatric age group. Pediatr Neurosurg 2009;45(3):181–184; doi: 10.1159/000218200 19440005

[6] Sinha S, Borkar S. Chronic calcified extradural hematoma in a child: Case report and review of literature. Indian Journal of Neurotrauma 2008;5(1):51–52.

[7] Gutowski P, Meier U, Rohde V, et al. Clinical outcome of epidural hematoma treated surgically in the era of modern resuscitation and trauma care. World Neurosurg 2018;118:e166–e174. 29959068 10.1016/j.wneu.2018.06.147

[8] de Oliveira Sillero R, Zanini MA, Gabarra RC. Large chronic epidural hematoma with calcification: A case report. J Trauma 2008;64(6):1619–1621; discussion 1621. 17554219 10.1097/01.ta.0000209403.56867.09

[9] Bullock MR, Chesnut R, Ghajar J, et al. Surgical Management of Acute Epidural Hematomas. Neurosurgery 2006;58(suppl_3):S2-7–S2-15; doi: 10.1227/01.Neu.0000210363.91172.A8 16710967

[10] Kotil K, Akcetin MA. Asymptomatic chronic ossified epidural hematoma in a child: A rare entity. Ulusal Travma Ve Acil Cerrahi Dergisi = Turkish Journal of Trauma & Emergency Surgery: TJTES 2006;12(2):164–166. 16676258

[11] Choudhary G, Singh R, Boparai A, et al. Epidural hematoma with ossification: Two case reports. PJR 2012;22(3).

[12] Bayri Y, Ulaş AK, UlUs A, et al. Iatrogenic chronic calcified/ossified epidural hematoma: Case report. Journal of Nervous System Surgery 2009;2(2):91–94.

[13] Nagane M, Oyama H, Shibui S, et al. Ossified and calcified epidural hematoma incidentally found 40 years after head injury: Case report. Surg Neurol 1994;42(1):65–69. 7940099 10.1016/0090-3019(94)90252-6

[14] Iwakuma T, Brunngra C. Extradural ossification following an extradural hematoma—case report. J Neurosurg 1974;41(1):104–106. 4210068 10.3171/jns.1974.41.1.0104

[15] Chang JH, Choi JY, Chang JW, et al. Chronic epidural hematoma with rapid ossification. Childs Nerv Syst 2002;18(12):712–716. 12483357 10.1007/s00381-002-0664-2

[16] Yu DK, Heo DH, Cho SM, et al. Rapidly calcified epidural hematoma in a neonate. J Korean Neurosurg Soc 2008;44(2):98–100. 19096702 10.3340/jkns.2008.44.2.98 PMC2588337

[17] Wong CH, Foo CL, Seow WT. Calcified cephalohematoma: Classification, indications for surgery and techniques. J Craniofac Surg 2006;17(5):970–979; doi: 10.1097/01.scs.0000229552.82081.de 17003628

[18] Chen J, Hu X, Yang L, et al. Bilateral large chronic ossified epidural hematoma after ventriculoperitoneal shunt: A special case report and treatment management. International Journal of Clinical and Experimental Medicine 2016;9(7):14515–14522.

[19] Turgut M, Akhaddar A, Turgut AT. Calcified or Ossified Chronic Subdural Hematoma: A Systematic Review of 114 Cases Reported During Last Century with a Demonstrative Case Report. World Neurosurg 2020;134:240–263; doi: 10.1016/j.wneu.2019.10.153 31682989

[20] Akhaddar ALI, Boulahroud O. Ossified chronic epidural hematoma of the posterior fossa. Pan African Medical Journal 2015;20. 10.11604/pamj.2015.20.238.3954 PMC4919671 27386034

[21] Kumar R, Mittal R. Posttraumatic chronic ossified extradural hematoma: a rare case report. Romanian Neurosurgery 2014:358–360.

[22] Miyazaki Y, Akagawa S. Calcified Epidural Hematoma. Neurol Med Chir(Tokyo) 1968;10:253a–253a; doi: 10.2176/nmc.10.253a

[23] Bishnoi S, Bishnoi I, Gahlawat N, et al. Ipsilateral Two Spontaneous Chronic Calcified Epidural Hematoma. Asian J Neurosurg 2019;14(3):1048–1049; doi: 10.4103/ajns.AJNS_199_17 31497163 PMC6703056

[24] Cambria S, Marra GA, Di Perri R, et al. Ossified epidural hematoma. Report of a case with epilepsy. J Neurosurg Sci 1985;29(3):285–288. 3831274

[25] Datta SG, Sharma C. Calcified Epidural Hematoma in an Adult Patient: A Case Report. Indian Journal of Neurotrauma 2016;13(03):171–173; doi: 10.1055/s-0036-1597942

[26] Dawar P, Phalak M, Sinha S, et al. Same side double chronic calcified epidural hematoma: case report and review of literature. Neurol India 2013;61(2):195–197; doi: 10.4103/0028-3886.111157 23644336

[27] Djoubairou BO, Gazzaz M, Dao I, et al. Chronic calcified extradural and subdural hematoma following a ventriculoperitoneal shunt placement. Neurol India 2015;63(2):282–283; doi: 10.4103/0028-3886.156316 25948009

[28] Agrawal VM, Giri P. Dual Chronic Ossified Epidural Hematomas Presented with Seizures 23 Years after Head Injury in an Adult Male: Case Report and Literature Review. Indian Journal of Neurotrauma 2018;15(01):041–042; doi: 10.1055/s-0037-1616032

[29] Han SR. Conservative management of a rapidly calcifying epidural hematoma in a young male patient. Interdisciplinary Neurosurgery 2015;2(4):183–185; doi: 10.1016/j.inat.2015.08.003

[30] Kawata Y, Kunimoto M, Sako K, et al. Ossified epidural hematomas: report of two cases. No Shinkei Geka. Neurological Surgery 1994;22(1):51–54. 8295702

[31] Kia-Noury M, Wiedenmann O. [Intracranial calcifications in epidural hematoma]. Neurochirurgia (Stuttg) 1963;6:33–39; doi: 10.1055/s-0028-1095426 14032444

[32] Kim DY, Jung JH, Kim DY, et al. Rapid Ossification of Epidural Hematoma in a Child: A Case Report. Korean J Neurotrauma 2014;10(2):152–154; doi: 10.13004/kjnt.2014.10.2.152 27169055 PMC4852624

[33] Leclercq TA, Rozycki T. Chronic calcified epidural hematoma in a child. Rhode Island Medical Journal 1979;62(3):97–99. 285450

[34] Mathuriya SN, Kak VK, Banerjee AK. Ossified epidural haematoma. Report of two cases. Clin Neurol Neurosurg 1989;91(3):269–272; doi: 10.1016/0303-8467(89)90124-8 2548795

[35] Matsumoto T, Nagai H, Sugiyama N, et al. [Chronic epidural hematoma. Report of two cases]. Neurol Med Chir (Tokyo) 1985;25(4):289–293; doi: 10.2176/nmc.25.289 2412152

[36] Mishra SS, Satapathy MC, Senapati SB. Shunt site chronic calcified extradural hematoma: An avoidable complication. J Pediatr Neurosci 2014;9(2):166–168; doi: 10.4103/1817-1745.139342 25250078 PMC4166845

[37] Nitta T, Hatashita S, Koga N, et al. A case of calcified epidural hematoma. Clinical Radiography 1984;29(5):603–605. 6482040

[38] Parkinson D, Reddy V, Taylor J. Ossified epidural hematoma: case report. Neurosurgery 1980;7(2):171–173; doi: 10.1227/00006123-198008000-00011 6775241

[39] Roganović Z, Zorić L, Antić B, et al. Ossified chronic epidural hematoma. Military-Medical and Pharmaceutical Review 1992;49(1):54–56. 1595234

[40] Sakai N, Yamamori T, Tanemura H, et al. Calcified epidural hematoma-report of a case incidentally found 16 years after head injury. No Shinkei Geka. Neurological Surgery 1977;5(2):163–167. 557736

[41] Sakurai T, Adachi S, Hayashi T, et al. A case of ossified epidural hematoma. Jpn J Neurosurg 1998;7(3):187–191.

[42] Schumacher M, Oldenkott P, Peiffer J, et al. [Unusual radiological and clinical findings in a calcified chronic extradural haematoma (author's transl)]. Neurochirurgia (Stuttg) 1982;25(1):1–6; doi: 10.1055/s-2008-1053945 7110482

[43] Erdogan B, Sen O, Bal N, et al. Rapidly calcifying and ossifying epidural hematoma. Pediatr Neurosurg 2003;39(4):208–211; doi: 10.1159/000072473 12944702

[44] Seyithanoglu H, Guzey FK, Emel E, et al. Chronic ossified epidural hematoma after ventriculoperitoneal shunt insertion: a case report. Turkish Neurosurgery 2010. 10.5137/1019-5149.JTN.2231-09.1 20963703

[45] Siedschlag WD, Schulz MR. Über ein verkalkendes chronisches epidurales Hämatom. Zentralbl Neurochir 1982;43:159–163. 7124192

[46] Trivedi A, Hiran S. Calcified epidural hematoma in pediatric age group: a report of two cases. Journal of Neurosciences in Rural Practice 2010;1(2):89. 21808510 10.4103/0976-3147.71716 PMC3139356

[47] Trodi NI, Ben Salem D, Mourier KL, et al. [Chronic calcified extradural hematoma]. J Neuroradiol 2007;34(1):69–71; doi: 10.1016/j.neurad.2007.01.005 17316799

[48] Whisler WW, Voris HC. Ossified epidural hematoma following posterior fossa exploration. Report of a case. J Neurosurg 1965;23(2):214–216; doi: 10.3171/jns.1965.23.2.0214 4954113

[49] Yeh CH, Wang CS, Yeh TC, et al. Ossified epidural hematoma. J Emerg Med 2015;48(2):43–44. 25453854 10.1016/j.jemermed.2014.09.031

[50] Yoshida N, Iseki H, Amano K, et al. A case with postoperative calcified epidural hematoma. No Shinkei Geka 1985;13(11):1199–1203. 4088442

[51] Zhang W, Zhang WEI, Gao Z, et al. Gigantic ossified chronic epidural haematoma and contralateral postoperative subdural haematoma: A case report and literature review. Br J Neurosurg 2015;29(1):85–86; doi: 10.3109/02688697.2014.952267 25174522

[52] Choudhary G, Singh R, Boparai A, et al. Epidural hematoma with ossification: Two case reports. PJR 2016;22(3).

[53] Jain S, Sundar I, Sharma V, et al. Chronic ossified extradural hematoma on the opposite side of the ventriculoperitoneal shunt procedure: A rare case report. Saudi Journal for Health Sciences 2012;1(3):159–161; doi: 10.4103/2278-0521.106087

[54] Mehra S, Tripathi AK, Singh A, et al. Calcified Extradural Hematoma with overlying Subcutaneous Swelling: A Rare Case Report. IOSR Journal of Dental and Medical Sciences (IOSR-JDMS) 2017.

[55] Sheyin J, Chom N, D, et al. Unusual Presentation of Epidural Haematoma with Ossified Border on Computed Tomography Scan. Nigerian Journal of Basic and Clinical Sciences 2021;18(2):156–158; doi: 10.4103/njbcs.njbcs_22_20

[56] Banga MS, Eep BV, Dixit S. Chronic Epidural Hematoma: Still a Rare Entity? Indian Journal of Neurotrauma 2021;19(01):023–028.

[57] Kanikomo D, Diallo M, Tokpa A, et al. Intracranial Calcified Extradural Hematoma about a Case. Ojmn 2023;13(02):69–73.

[58] D’Andrea M, Mongardi L, Cultrera F, et al. Calcified Epidural Hematoma after Conservative Treatment of Acute Epidural Hematoma in the Pediatric Population: A Systematic Review. Pediatr Neurosurg 2022;57(6):389–395; doi: 10.1159/000527241 36167051

[59] Pang D, Horton JA, Herron JM, et al. Nonsurgical management of extradural hematomas in children. J Neurosurg 1983;59(6):958–971. 6631518 10.3171/jns.1983.59.6.0958

[60] Pappamikail L, Rato R, Novais G, et al. Chronic calcified subdural hematoma: Case report and review of the literature. Surg Neurol Int 2013;4:21; doi: 10.4103/2152-7806.107548 23493910 PMC3589848

[61] Niwa J, Nakamura T, Fujishige M, et al. Removal of a large asymptomatic calcified chronic subdural haematoma. Surg Neurol 1988;30(2):135–139. 3400041 10.1016/0090-3019(88)90099-7

[62] Oda S, Shimoda M, Hoshikawa K, et al. Organized chronic subdural haematoma with a thick calcified inner membrane successfully treated by surgery: A case report. Tokai J Exp Clin Med 2010;35(3):85–88. 21319032

