# Supplementary material for: Ossification of Cranial Epidural Hematomas: A Systematic Review of Management Strategies and Presentation of an Illustrative Case
Source: Neurotrauma Rep. 2024 Aug 22;5(1):787–99. doi: 10.1089/neur.2024.0065 (PMC11342046; doi:10.1089/neur.2024.0065)
Supplement: Supplementary Appendix S1 [file neur.2024.0065_appendix.pdf]

	Author(s), year of publication	Type of article	Age/ Sex (F/M)	Co-morbidities, coagulation status	Time between trauma/ surgery and first diagnostic of calcified/ ossified EDH	Previous trauma or surgery	Kind of trauma/ type of surgery	Imaging after Initial trauma/surgery? (yes/no)	Treatment of calcified/ossified EDH	Surveillance or rapid surgery after diagnosis of calcified/ossified EDH? Evolution	Symptomatology/ neuropsychological exam	imaging	EEG	Localization/ Volume/ intraoperative findings/ histology
1	**Kotil and Akçetin, 2006**	CRP/1	6/M	- no metabolic, endocrinological or chronic disease<br>- routine biologic exam without pathological findings<br>- extended studies (protein S and C deficiency, factor V Leiden, Antithrombin III) normal	3 weeks	trauma	head trauma, not specified	no	surgery, craniotomy	- surgery without urgence<br>- surgery due to potential complications	- asymptomatic,<br>- no neuro-psychological exam	- skull X-ray: no fracture<br>- scan: ossified hematoma	no	- left frontal<br>- volume: 4x 3,5x 2,5 cm<br>- osseous tissue 7mm thick<br>- no important mass effect<br>- intraoperative: adherend to dura mater, removed with dura, no capsule<br>- no histology<br>- no complications postoperative
2	**Akhaddar and Boulahroud, 2015**	CRP/1	15/M	routine biologic parameter normal	2 months	trauma	- stone-throwing<br>- no loss of consciousness	no:<br>- trauma neglected<br>- no physician visited after trauma	surgery, suboccipital craniotomy	surgery without urgence	- mild headache<br>- cerebellar syndrome with progressive dizziness<br>- vomiting since two weeks<br>- no neuropsychological exam	- scan: hypodensity in center with calcified wall<br>- MRI: center liquid and ring enhancement after gadolinium	no	- posterior fossa, left cerebellar<br>- volume: not specified<br>- slight mass effect<br>- intraoperative: thick hard wall adherend to the dura<br>- histology of wall: large area of ossification<br>- no complications postoperative
3	**Banga et al., 2021**	Case series (1 case of ossified EDH)	30/M	mentally retarded	unclear	trauma, multiple falls	-	no	surgery, craniotomy	surgery without urgence	- progressive weakness of all limbs since 6 months (M3/5)<br>- no neuropsychological exam	scan	no	2 localizations:<br>- left parietal (most calcified, less mass effect)<br>- left frontal (calcified wall, important mass effect)<br>- volume: not specified<br>- intraoperative: dura calcified, partially resected (adherence to dura)<br>- no histology<br>- no complications postoperative
4	**Miyazaki and Akagwa, 1968**	CRP/1	6/M	routine biologic exam without pathological findings	4 months	trauma	accident, struck by a taxi	yes:<br>- X-ray without pathological findings	surgery, craniotomy	surgery without urgence	- initial asymptomatic<br>- later (7 weeks) headache - hypesthesia upper extremity<br>- no neuropsychological exam	- X-ray<br>- ventriculography<br>- angiography	no	- right parietal<br>- volume: 8x9x3cm, liquid 30 cc<br>- intraoperative findings not specified<br>- mass effect<br>- no histology
5	**Bishnoi et al., 2018**	CRP/1	12/M	- tubercular meningitis and hydrocephalus at age of 4 (no VP-shunt at that time, refused by parents)<br>- no coagulation disorder	unclear	- unclear, no history of trauma<br><br>- bike fall 2 days ago leading to imaging	unclear	- no history of trauma<br><br>- diagnostic of calcified/ossified EDH in scan after acute trauma	conservative of both calcified and acute hematoma	- control scan after 1 and 3 months: after 3 months reduction of hematoma parietal, frontal hematoma disappeared, calcification stable	- headache and vomiting following fall from bike 2 days before<br>- hematoma with calcification asymptomatic (age unclear), diagnostic due to head trauma, symptoms due to acute component<br>- no neuropsychological exam	scan/ MRI: acute on chronic calcified, EDH, max. thickness 4,7 cm + small frontal subdural hematoma, blood of different age, hydrocephalus	no	- right parietal<br>- maximal thickness of hematoma 4,7 cm<br>- reduction to 2,4 cm under conservative treatment<br>- mass effect
6	**Cambria et al., 1985**	CRP/1	24/M	routine biologic exam without pathological findings	- 15 months<br>- Surgery after 4,5 years	trauma	trauma, loss of consciousness for several minutes	- no initial imaging,<br>- surveillance,<br>- rapid improvement - discharge after 10 days	surgery, craniotomy + lobectomy + resection epileptogenic focus (electrocorticograph ic recording)	- six month after diagnostic two seizures, antiepileptics were introduced,<br>- after 15 months seizure again: image (X-ray showed right frontal opacity),<br>- in the following 4,5 years episodes of seizures/ almost epileptic status	- surgery due to increasing epileptic seizures<br><br>- no neuropsychological exam	- first skull X-ray after 15 month: right frontal opacity (calcification),<br>- X-ray ad admission: right frontal calcification<br>- angiography right carotid after 4,5 years later without pathology<br>- still ossification in skull X-ray	EEG: right frontocent ral epileptic focus	- right frontal<br>- volume: not specified<br>- intraoperative: bone flap thickened in central and lower parts<br>- good evolution, reducing and stop of antiepileptics during next years<br>- no complications postoperative, normal life
7	**Chang et al., 2002**	CRP/1	13/F	- healthy<br>- blood analyses normal	- day 30: mild calcification,	trauma	motor vehicle accident, involved as pedestrian	yes:<br>- initial scan without	- surgery,<br>- but initial surveillance	- surveillance,	- persisting headache, nausea vomiting<br>- no neuropsychological exam	- scan day 16 due to mild headache: sub-acute EDH,	no	- right frontal<br>- slight mass effect, no midline shift<br>- volume: 3 x 4.5 cm, ossification 2-3mm, capsule

					- day 60:<br>progress of<br>calcification			pathological<br>findings, only<br>scalp swelling<br>parietal	because absorption<br>was expected,<br>- craniotomy at day<br>73	- scan day 16 due to mild<br>headache (first symptoms): sub-<br>acute EDH,<br>- scan day 30: beginning of<br>calcification<br>- scan day 60: calcification<br>progressed, volume stable		- scan day 32:<br>beginning of<br>calcification,<br>- scan day 60:<br>calcification<br>progressed, volume<br>stable		- intraoperative: capsule, bone adherent to dura<br>yellowish to dark brown liquified hematoma<br>- histology: thick fibrotic/ collagenous hematoma capsule,<br>macrophage in hematoma containing hemosiderin, no<br>calcium deposit, bone totally ossified
8	**Chen et al.,<br>2016**	CRP/1	35/M	- not specified<br>- hydrocephalus	- first<br>calcification seen<br>after 2 years,<br>- surgery after 19<br>years	surgery	- VP-shunt temporo-<br>occipital due to<br>hydrocephalus	- scan 3 days<br>postoperative<br>showed bilateral<br>epidural<br>hematoma<br>- follow up by<br>scan,<br>- 2 years first<br>partly<br>calcification<br>visible,<br>- surgery after 19<br>yrs	- surgery,<br>craniotomy bilateral<br>- complicated by<br>intracerebral<br>bleeding and<br>hydrocephalus<br>postoperative,<br>- new surgery, EVD<br>and VP-shunt,<br>- further evolution<br>good	surgery without urgence	- severe headache and<br>neurological symptoms since<br>two weeks<br>- neurological examination<br>revealed normal findings<br>except walking disorder and<br>bilateral paresis<br><br>- no neuropsychological exam	- scan: calcified<br>hematoma bilateral<br>(right frontal, left<br>parietal)	no	2 localizations:<br>- right frontal, left parietal<br>- intraoperative: attached to dura, dissection was possible<br>- volume: not specified<br>- mass effect<br>- histology: thick fibrotic hematoma capsule, small<br>hematoma content. Ossification, calcification and fibro-<br>collagenous areas were seen in the hematoma.<br><br>- complication: Intracerebral hematoma and<br>hydrocephalus, surgery for hematoma, shunt
9	**Claiborne et<br>al., 2015**	CRP/1	2/F	not specified	several weeks	surgery	- fenestration of<br>arachnoid cyst right<br>middle fossa<br>- VP-shunt days later<br>due to epidural fluid<br>collection et CSF<br>subgaleal	yes:<br>- scan after some<br>days: bifrontal<br>epidural<br>hematoma +<br>subgaleal<br>collection	surgery, craniotomy<br>ad cranioplasty	surgery without urgence	- calvarial skull deformity, -<br>headache,<br>- scan was done due to<br>symptoms and new head<br>trauma<br><br>- no neuropsychological exam	scan: ossified epidural<br>hematoma bilateral	no	- bifrontal<br>- mass effect left frontal<br>- volume: not specified
10	**Datta and<br>Sharma, 2016**	CRP/1	31/M	routine biologic<br>exam without<br>pathological<br>findings	1 month	trauma	head trauma with loss<br>of consciousness for 15<br>min	yes:<br>- scan day 0 after<br>admission: aEDH<br>left frontal,<br>- 7 days after<br>trauma: aEDH<br>left frontal stable	conservative	- EDH stable at scan day 7<br>- scan day 12 due to persisting<br>headache: EDH decreasing<br>- scan before discharge 1 month<br>after trauma: EDH smaller, thin<br>layer of calcification after 1<br>month<br>- no follow up scan after<br>discharge	- persisting headache in the<br>beginning, after that<br>asymptomatic<br><br>- no neuropsychological exam	- EDH stable in scan<br>day 7<br>- scan day 12 due to<br>persisting headache:<br>EDH decreasing<br>- scan before discharge<br>1 month after trauma:<br>EDH smaller, thin layer<br>of calcification<br>- no follow up scan<br>after discharge	no	- left frontal<br>- no mass effect
11	**Dawar et al.,<br>2013**	CRP/1	18/M	routine biologic<br>exam without<br>pathological<br>findings	20 days	trauma	fall from 2nd floor, no<br>loss of consciousness	scan: aEDH,<br>initial<br>conservative<br>cause patient<br>was stable	surgery, craniotomy	emergency	- altered consciousness,<br>deterioration within 6h<br>before arrival at hospital,<br>mydriasis unilateral<br><br>- no neuropsychological exam	scan: EDH right frontal<br>and parietal with<br>hyperdense layer	no	2 localizations:<br>- right frontal and parietal<br>- osseous layer 3mm attached to the dura without plane
12	**de Oliveira<br>et al, 2008**	CRP/1	12/M	routine biologic<br>exam without<br>pathological<br>findings	1 month	trauma	- fall from height<br>- no loss of<br>consciousness	no	surgery, craniotomy	prompt surgery but without<br>urgences	- left parietal headache and<br>vomiting<br><br>- no neuropsychological exam	- skull X-ray: no<br>fracture,<br>- scan: EDH mixed<br>density, enhancement<br>of capsule, calcification	no	- left parietooccipital<br>- volume: 8x5x7cm<br>- mass effect, midline shift<br>- intraoperative: thick encapsuled hematoma, adherent to<br>the dura, thin layer covering the brain was left.<br>- hematoma was fluid with a clot, capsule consisted of<br>fibroblasts and collagenous fibers with calcium deposit
13	**Djoubairou et<br>al., 2018**	CRP/1	22/M	routine biologic<br>exam without<br>pathological<br>findings	20 years	surgery	- VP-shunt in childhood<br>for congenital<br>hydrocephalus,<br>- no history of trauma	no follow up<br>after shunt<br>implantation	- conservative,<br>symptomatic<br>therapy,<br>- regression of<br>symptoms after 5<br>days of pain<br>medication,<br>- clinical follow up,<br>- no further imaging	-	- headache, vomiting,<br>- learning difficulty since<br>childhood<br>- no neuropsychological exam	X-ray: calcification,<br>scan: calcified EDH and<br>SDH,<br>MRI confirmed<br>diagnostic	no	- parietooccipital right<br>- no mass effect
14	**Agrawal et<br>Giri, 2018**	CRP/1	35/M	routine biologic<br>exam without<br>pathological<br>findings	23 years	trauma	fall from a tree	no	- conservative,<br>- patient refused<br>EEG and surgery for<br>financial reasons,	-	- seizures, headache<br>- no neuropsychological exam	scan	no	2 localizations<br>- right frontal, left parietal

									- antiepileptics due to seizure and follow up					
**15**	**Han, 2015**	CRP/1	20/M	routine biologic exam without pathological findings	6 months	trauma	fall	- scan: EDH left frontal+ fracture,<br>- control scan after 24h: increased EDH,<br>- MRI after 1 week: increased hematoma,<br>- scan two weeks: hyperdense layer,<br>- scan after 6 month: liquid decreased but hyperdense layer increased<br>- scan 12 months: almost complete absorption of EDH with remaining calcification	- conservative because only mild headache,<br>- surveillance,<br>- scan after one year: resorption of the liquid part, remaining calcification	-	asymptomatic	scan	no	- left frontal<br>- calcification appeared after two weeks, follow up 1 year<br>- mild mass effect on the lateral ventricle in MRI
**16**	**Iwakuma and Brunngraber, 1974**	CRP/1	33/M	epilepsy	1 month	trauma	fell during seizure	yes:<br>skull X-ray: skull fracture left frontal	surgery, craniotomy	without urgences	- mental disorder, confusion, irritability, lack of concentration and disorientation	- X-ray: hyperdense layer<br>- angiography: arteria cerebral media displaced, avascular area	Yes:<br>discrete theta waves left frontal without paradoxal discharge	- left frontotemporal<br>- volume 7x6x2 cm<br>- between hematoma and dura 0,5 cm spongy calcified mass adherent to dura and capsule<br>- along the outer margin of the calcified plate which was in contact with the inner table of the skull, there were fine bony spicules.<br>- the capsule was 5mm thick and histologically consistent of fibroblasts and collagen fibers. Microscopic dissection of the bony mass made after decalcification revealed a typical bone structure.<br>- discharge after 12 days but still confused
**17**	**Kawata et al., 1994**	CRP/2	Case 1: 9/M<br><br>Case 2: 12/M	Case 1: not specified<br><br>Case 2: not specified	Case 1: 3 months<br><br>Case 2: 12 days	Case 1: trauma<br><br>Case 2: trauma	- both traffic accident<br><br>- Case 2: loss of consciousness for 10 min and seizure, no symptoms ad admission at hospital	Case 1:<br>- scan: fracture parietal and thin aEDH treated conservatively, control after 3 months<br><br>Case 2:<br>- X-ray: skull fracture,<br>- scan: 1cm aEDH treated initial conservatively, control 12 days	Case 1: surgery, craniotomy<br><br>Case 2: surgery, craniotomy	prompt surgery but without urgences	Case 1: no symptoms, diagnostic in control scan<br><br>Case 2: asymptomatic, diagnostic in control scan	Case 1:<br>- X-ray and scan: fracture right parietal + EDH<br><br>Case 2:<br>- scan: calcification after 12 days – membrane calcified, volume stable	Case 1: no<br>Case 2: no	Case 1:<br>- right parietal<br>- calcification capsule 2-3mm<br>- slight mass effect<br>- dura adherent<br>- histology: bone in fibrous capsule adherent to dura<br><br>Case 2:<br>- left parietal<br>- slight mass effect<br>- histology: calcification and osteocytes
**18**	**Kia-Noury and Wiedenmann , 1963**	CRP/1	10/F	routine biologic exam without pathological findings	1 month after burr hole for evacuation of EDH	trauma + surgery for aEDH	- 05.04.61: head trauma, fall 2 m, explorative burr holes J0 negative.<br>- 06.04.61: girl comatose, transfer to other hospital with mydriasis<br>- day 19 (24.04): burr hole epidural to drain hematoma,<br>- craniotomy the 26.05.23:	- day 1: X-ray : no fracture,<br>- 12.04.61 arteriography: lesion parietooccipital	craniotomy	prompt surgery but without urgences	headache, hyperreflexia , occasional vomiting	- X-ray<br>- angiography	yes:<br>- focal signs, epileptic focus parietal/occipital	- left parietal<br>- volume not specified<br><br>- no intraoperative description<br>- no histology

							intraoperative calcification							
**19**	**Kim et al., 2015**	CRP/1	5/M	routine biologic exam without pathological findings	14 days	trauma	traffic accident	yes:<br>- X-ray skull: parietal fracture<br>- scan: left parietal EDH	conservative<br><br>parents refused operation	- scan day 7: EDH slightly increased in volume<br>- scan day 14: EDH size slightly decreased but high hyperdense layer<br>- epidural calcification was growing in thickness during first 2-3 months<br>- scan six month after trauma: EDH fully absorbed, calcified lesion merged to inner table of skull	- slight headache at beginning, later asymptomatic	no	no	- left temporoparietal<br>- no info about volume<br>- slight mass effect
**20**	**Leclercq and Rozycki, 1979**	CRP/1	3/M	routine biologic exam without pathological findings	3-4 months	trauma	in June 1975 fall with loss of consciousness for a few minutes	X-ray: left parietal fracture and huge subgaleal hematoma	surgery, craniotomy frontoparietal	immediate wo urgences	- in october the patient fell again<br>- the teacher realized that he was clumsy and had the tendency to lose the balance<br>- gait broad base	X-ray: known fracture with hyperdense layer scan	yes: EEG unremarkable	- left frontotemporal<br>- no mass effect<br>- intraoperative: brownish-greenish membrane encapsulating hematoma, membrane 2.5-3 mm<br>- no volume info<br>- histology: fibrillary organization with areas of fibroblastic proliferation, and new capillary formation. Hemosiderin was present in macrophages. Small areas of newly formed ossification were identified
**21**	**Lee et al., 2014**	CRP/1	21/F	no, blood exam normal	17 days (first calcification)	trauma	- struck by motor vehicle, no loss of consciousness	yes:<br>- scan: fracture right frontal,<br>- scan day 11 after trauma: EDH<br>- scan day 17: first calcification	conservative treatment	- no surgery,<br>- follow up with scan day 11 and 17 ,1, 1.5, 2,3, 5.5 and 9 months after trauma,<br>- EDH disappeared, calcification increased	mild headache		no	- right frontal<br>- no mass effect<br>- no volume info
**22**	**Mathuriya et al., 1989**	CRP/2	Case 1: 35/M<br><br>Case 2: 29/M	Case 1: routine biologic exam without pathological findings<br><br>Case 2: routine biologic exam without pathological findings	Case 1: 3 months<br>Case 2: 5 weeks	Case 1: surgery, VP-shunt for hydrocephaly<br><br>Case 2: surgery, VP-shunt for hydrocephalus	Case 1: surgery, VP-shunt for hydrocephaly<br><br>Case 2: surgery, VP-shunt for hydrocephaly	-	Case 1: surgery<br>Case 2: surgery	immediate wo urgences	Case 1: admission due to loss of consciousness, seizure, hemiparesis<br><br>Case 2: seizure, confusion	Case 1:<br>- angiography left carotid: epidural mass,<br>- X-ray: normal<br><br>Case 2:<br>- scan: cEDH with calcification, hydrocephalus due to shunt dysfunction	Case 1: yes, bilateral and temporal slowing<br><br>Case 2: no	Case 1:<br>- right temporoparietal<br>- 3 cm thick<br>- adherent to dura<br>- contained thick fibrocollagenos mass, 0,5 ml brownish fluid<br>- histology: normal and new bone in the inner shell. Organized mass was composed of collagen tissue with calcification<br><br>Case 2:<br>- right parietal<br>- mass effect<br>- 50ml liquid, inner bony shell over the dura covered by granulations<br><br>- postoperative: complicated by shunt-infection and ventriculitis
**23**	**Matsumoto et al., 1985**	CRP/1	15/F	routine biologic exam without pathological findings	36 days	trauma	fall from motorcycle, loss of consciousness for 30 min	no	surgery	immediate wo urgences	mild headache	scan	no	- left parietal<br>- no Info volume<br>- mass effect<br>- histology osteoblasts and osteocytes in calcified part on side of dura, neovascularization
**24**	**Mishra et al., 2014**	CRP/1	18/M	- hydrocephalus,<br>- coagulation exam normal	3 months	surgery	VP-shunt right parietal	-	surgery	immediate wo urgences	- headache<br>- sensory alteration since 10 days	scan: chronic calcified hematoma	no	- right parietal<br>- mass effect<br>- no info volume
**25**	**Nagane et al., 1994**	CRP/1	57/M	- hypertension (brain MRI for screening of hypertension)<br><br>- blood examination negative for coagulation	40 yrs	trauma	- head trauma during baseball match at the age of 17<br>- loss of consciousness<br>- 3 days coma<br>- numbness in the hand<br>- release of symptoms after 2 weeks	skull X-ray: fracture parietal	surgery, craniotomy due to mass effect and also to differentiate from epidermoid, abscess, fibrous dysplasia et osteitis fibrosa	immediate but without urgences	- incidental<br>- MRI due to hypertonia diagnostic<br>- slide heaviness of head,<br>- no deficit	- X-ray: oval radiopaque lesion with a curving double line<br>- scan confirmed isodense intracranial mass whose inner surface was bordered with a thick (max 8mm) partially outlined	no	- left parietal<br><br>- volume 7x4,5x3cm<br>- mass effect<br><br>- intraoperative: mass fused with skull. The inner surface of the shell, uniformly ossified was easily freed from the dura by blunt dissection, tight adhesion at temporal edge.

				disorder and metabolic problems								hyperdense layer. Calvarium over the outer surface of the mass had normal structure at the margin but become thinner in the central region<br>- MRI<br>- Internal/external carotid angiography: avascular lesion		Yellowish brown gelatinous hematoma, only partially liquified within the shell<br><br>- histology: hematoma capsule consisted of collagen fibers and fibroblast with a marked calcification inside the inner and outer shells. A lamellar bone layer was continually formed between dura and calcified inner capsule. Ossified shell fused with the outer shell consisting of the normal calvarium at the margin
26	**Nitta et al., 1984**	CRP/1	73/M	no comorbidities	50 yrs	trauma	- after trauma hemiparesis and changings of consciousness	no	conservative because asymptomatic since 50 yrs	-	- asymptomatic<br>- imaging due to new cranial trauma	- X-ray: calcification<br>- scan<br>- angiography	no	- left parietal<br>- mass effect<br>- no info volume
**27**	**Parkinson et al., 1980**	CRP/1	38/F	- astrocytoma at 20 yrs<br>- retinitis pigmentosa	18 yrs	surgery	- resection of astrocytoma 18 years before	no	surgery, craniotomy	immediate but without urgences	- memory problems since 6 weeks<br>- some headache, personality changing	- X-ray<br>- angiography	no	- left parietal<br>- no volume info<br>- inner surface freely dissected from dura, no line demarcation between calvarium and lesion
28	**Roganovic et al., 1992**	CRP/1	31/M	no	1 yr	trauma	no	no	surgery	- initial conservative<br>- diagnostic 1 year after trauma due to headache, dizziness<br>- surgery because of seizure	- headache, dizziness<br>- paresthesia right hand<br>- surgery due to seizure	- X-ray<br>- scan<br>- angiography	no	- left temporal<br><br>- slight mass effect<br>- volume: 6x3cm<br>- ossification: 1mm<br>- histology: encapsuled hematoma
29	**Sakai, 1977**	CRP/1	55/M	- hypertonia<br>- no other comorbidities	16 yrs	trauma	no	no	surgery, craniotomy		- headache<br>- changing of consciousness<br>- temporary speech disorder	- X-ray<br>- angiography	no	- left parietal<br><br>- volume: 10x7x1,2cm<br>- ossification: 12mm
30	**Sakurai et al., 1998**	CRP/1	14/f	no	8 months	trauma	struck by a golf club frontal	- X-ray: no fracture<br>- scan: right frontal aEDH treated conservatively, 10 days surveillance	surgery, craniotomy because resorption no more likely	Surgery without urgence	asymptomatic	- control scan 8 month: calcification and augmentation	yes: pathologic	- right frontal<br>- slight mass effect<br>- histology: thick fibrous connective tissue with inflammatory cells in the outer membrane and ossification with lamellar bone layers in the inner membrane
31	**Schumacher, 1982**	CRP/3	Case 1: 20/M<br><br>Case 2: 11/F<br><br>Case 3: 30/M	Case 1: C2-abusus<br><br>Case 2: pubertas praecox<br><br>Case 3: C2-abusus	Case 1: 2 yrs<br><br>Case 2: 19 days<br><br>Case 3: 1 month	Case 1: surgery due to trauma<br><br>Case 2: surgery, EDH bilateral after Mayfield<br><br>Case 3: trauma	Case 1: surgery due to traumatic EDH left frontal<br><br>Case 2: VA-shunt, tumor resection III ventricle, aEDH bilateral after Mayfield<br><br>Case 3: not specified	Case 1: scan<br><br>Case 2: scan<br><br>Case 3:<br>- X-ray: normal, after one month calcification, progression after<br><br>Case 3: no image	Case 1: conservative<br><br>Case 2: surgery parietal<br><br>Case 3: surgery 6,5 after trauma	Case 1: no symptoms, new scan after new trauma<br><br><br>Case 2:<br>- scan day 9<br>- scan day 19: hyperdense membrane, immediate but without urgences<br><br>Case 3 : immediate but without urgences	Case 1: no symptoms, new scan after new trauma<br><br>Case 2: asymptomatic<br><br>Case 3: grand mal seizure, pain and hypesthesia trigeminus V1	Case 1:<br>- scan: EDH calcified with new brain edema and traumatic SAB<br><br>Case 2:<br>- scan day 9<br>- scan day 19: hyperdense membrane<br><br>Case 3:<br>- X-ray: oval calcification,<br>- X-ray 4 weeks later: increased calcification<br>- scan: calcification 1.5 cm<br>- scan after 7 weeks and 6.5 month: increasing calcification	Case 1: no<br>Case 2: no<br>Case 3: no	Case 1:<br>- left frontal, no mass effect, no info about volume<br><br>Case 2:<br>2 localizations<br>- frontal right, parietal left,<br>- no info about volume<br>- slight mass effect<br><br>histology: hematoma membrane with fibroblasts, vessels with islands of ossified tissue and osteoid tissue, thin layer of osteoblasts. No bony shells on the side of visceral membrane.<br><br>Case 3:<br>- frontal left<br>- no volume info<br>- slight mass effect<br>- intraoperative: adherent to skull, bony shells similar to calvarium<br>- histology: areas of normal and new bone in the inner shell with bony lamellas in parallel order to calvarium, with partly normal hematopoietic marrow, osteoblasts only on the visceral side
32	**Erdogan et al., 2003**	CRP/1	8/M	no metabolic or endocrinologic disease	10 days	trauma	fall from second floor 10 days before	Yes:<br>- scan: aEDH, -initial conservative	surgery, craniotomy	immediately in urgences (EDH bigger than in first scan and neurological deterioration)	- mild lethargy<br>- headache, nausea<br>- mild left hemiparesis,<br>- altered consciousness,	scan	no	- right parietal<br><br>- calcification 3mm<br>- slight mass effect

								treatment then neurological deterioration			- signs of elevated ICP,<br>- patient deteriorated progressively within 3h			- adhesion to dura, no capsule<br>- histology: organized hematoma with ossification and calcification. No inflammatory signs.
33	**Seyıthanoglu, 2010**	CRP/1	17/F	no	3 yrs	surgery	VP-shunt implantation due to triventricular hydrocephalus right temporooccipital	no	surgery, craniotomy	immediate but without urgences	increasing headache after 3 yrs of chronic headache	- X-ray- oval radiopaque lesion bifrontal<br>- scan	no	- bifrontal<br>- slight mass effect, no info volume<br>- Intraoperative: calcification adherent to dura<br>- thick organized, fibrocollagenous mass, 10 ml dark yellow fluid<br>- calcification/ ossification on dural side
34	**Siedschlag and Schulz, 1982**	CRP/1	16/F	no	58 days	surgery	- resection of choroid plexus papilloma of the VI. Ventricle<br><br>- trepanation right parietal for EVD	- control scan day 58, complication postoperative with cranial nerve palsy, tracheotomy, ventilation for 51 days<br>- day 15: seizure	surgery, craniotomy	immediate but without urgences	postoperative complication: day 15 seizure	- scan day 58 after surgery: hematoma right parietal	EEG non patho-logic	- parietal left,<br>- mass effect<br>- intraoperative: layer of calcification lying on the dura<br>- volume not specified<br>- no histology
35	**Trivedi and Hiran, 2010**	CRP/2	Case 1: 6.5/F<br><br>Case 2: 9/M	Case 1: not specified<br><br>Case 2: anemia, not specified	Case 1: 6 months<br><br>Case 2: 5 months	Case 1: surgery infratentorial<br><br>Case 2: trauma	Case 1: operation for a right cerebellar astrocytoma<br><br>Case 2: fall	Case 1: control scan 6 months<br><br>Case 2: no	Case 1: surgery,<br><br>Case 2: surgery	without urgences	Case 1:<br>- mild headache, mild cerebellar syndrome<br>- control scan 6 months after, incidental finding<br><br>Case 2:<br>- mild headache for 3 months<br>- walking disorder holocranial headache with dullness since 4 months, he was unable to walk properly since 3 months	Case 1:<br>- scan: chronic EDH with ossified layer 3-4 mm<br><br>Case 2: scan revealed a large calcified bilateral epidural hematoma with hypodensity at the center.	Case 1: no<br><br>Case 2: no	Case1:<br>- parietal left<br>- mass effect<br>- no info volume<br>- no histology<br>- Intraoperative: after the bone flap was removed, a yellowish, thick, elliptical capsule was noted. The capsule was easily dissected from the surrounding bone. The bisected hematoma capsule revealed a watery fluid. A newly formed bone at the inner border of the hematoma capsule was completely covering the underlying dura. The bone that was attached to the dura was 3 mm thick.<br><br>Case 2:<br>- Mass effect<br>- No info volume<br>- No histology<br>- bifrontal craniotomy was done and a large chronic hematoma involving the frontal region and liquefied altered blood with thick hard calcified walls, adherent to the duramater, was encountered. Plain was well preserved between the calcified wall and the duramater.
**36**	**Trodi et al., 2007**	CRP/1	36/M	- HIV stage CDC C3<br>- alcohol abuses	12 days	trauma	no	scan: aSDH frontal left, aEDH + contusion, conservative treatment	conservative	- day 12 after trauma already calcification,<br>- two years EDH smaller but calcification increased	- seizure after cranial trauma, after that asymptomatic	scan: calcified EDH after 12 days	no	- left frontal,<br>- thickness of calcification 10 mm,<br>- slight mass effect
**37**	**Wishler et al., 1964**	CRP/1	27/M	Hemangioblastoma, posterior fossa	14 month	surgery	- hemangioblastoma right cerebellar<br>- tapping of lateral ventricle right parietal during surgery	no	surgery, craniotomy	immediate but without urgences	- asymptomatic,<br>- admission due to backpain irradiating both legs	- X-ray: 6x8 cm ring of calcification<br>- angiography	no	- right parietal<br>- 6x8 cm, fibrotic sac 5x4x1 cm, 15 cc liquid right parietal<br><br>- bone shell became continuous with calvarium fibrotic sac<br>- Inner surface of bony shell with bone spicules adherent to dura
**38**	**Yeh et al., 2014**	CRP/1	8/F	ventricular anaplastic ependymoma	1 year	surgery	surgery for left ventricular anaplastic ependymoma	- post-operative EDH,<br>- asymptomatic,<br>- treatment conservative with observation	surgery, craniotomy	immediate but without urgences	- 1 year after surgery generalized seizure<br>- GCS 10<br>Hemiparesis<br>- neurologic examination, including cranial nerves and cerebellar examinations normal.	scan	no	- left parietal<br>- volume: 44x 30x 35 mm<br>- mass effect<br>- histology: mainly fibrotic tissue and sclerotic bony fragments
39	**Yoshida et al., 1985**	CRP/1	14/M	- pineal region tumor<br>- hirsutisms, coagulation	3 years	surgery	- ventriculoperitoneal shunt,	no	surgery	3 months later	headache	X-ray: calcification	no	- right parietal<br>- volume: 7.0 X 7.5 cm and 2.5 cm, 2mm thick<br>- capsule

				normal<br>- adrenogenital syndrome			- and radiotherapy for pinealoma 3 years before							
**40**	**Yu et al., 2008**	CRP/1	26 days/F	- no<br>- extended blood tests normal	25 days	birth trauma, caesarian section	- birth trauma,<br>- caesarian section due to delayed/ failed normal vaginal delivery	no	- surgery,<br>- small burr-hole trephination was performed and a single silastic drainage catheter was inserted	- immediate surgery	- mild scalp swelling after birth, especially prominent in the right frontal area<br><br>- on the 25th day after her birth, the baby presented with drowsiness and hypotonia following a generalized tonic-clonic seizure	- MRI<br>- scan<br><br>- scan after 2 weeks: EDH resolved, calcified layer still there, attached to the bone	No	- right frontal<br><br>- slight mass effect<br><br>- no info volume<br>- no histology
**41**	**Zhang et al., 2015**	CRP/1	21/F	- thrombocyto-penia purpura in infancy (age of 2 months)<br><br>- blood exam and coagulation normal	not clear, probably years	spontaneo us, unclear	-	-	surgery, craniotomy	-	- headache for years,<br><br>- during follow up after surgery: up mild hemiparesis<br>- no neuropsychological exam	scan: huge calcified hematoma	no	- right hemisphere<br>- important mass effect<br><br>- intraoperative: yellow mass, with ossified outer shell, hyperplasia of skull while the inner side of the shell was attached to the dura. Removal of ossified tissue was hart for the partly adhesion of the shell, dura and cortex<br><br>- histology: organized hematoma with calcification<br><br>- complication: 2 month later a chronic subdural hematoma contralateral had to be drained
**42**	**Choudhary et al., 2012**	CRP/2	Case 1: 30/M<br><br>Case 2: 26/M	Case 1: coagulation, metabolic and endocrinologic disorder ruled out<br><br>Case 2: no, hematological analysis normal	Case 1: 6 weeks<br><br>Case 2: 1 week	Case 1: trauma<br><br>Case 2: trauma	Case 1: traffic accident 4 years ago<br><br>Case 2: struck with a brick on his head, transient loss of consciousness	Case 1: initial scan: EDH + fracture left frontal,<br>- control scan after 2 and 6 weeks and 2 yrs.<br>- after 6 weeks first calcification<br><br>Case 2: scan after 1 week: EDH in resorption with hyperdense layer	Case 1: surgery, craniotomy 4 years after accident due to seizures<br><br>Case 2: conservative	Case 1: control scan after 2,6 weeks + 2 yrs.<br>- after 6 weeks first calcification,<br>- slow augmentation during years<br><br>Case 2: scan one week after trauma, fracture and EDH with hypertense layer	Case 1: seizure since 3 years (start 1 year after trauma)<br><br>Case 2: no symptoms	Case 1: scan<br><br>Case 2: scan	Case: 1 yes: no pathology<br><br>Case 2: no	Case 1:<br>- left frontal<br>- slight mass effect<br><br>- no info volume<br>- no histology<br><br>Case 2:<br>- left frontal<br>- no mass effect<br>- no info volume<br>- no histology
**43**	**Jain et al, 2012**	CRP/1	21/M	- no<br>- coagulation profile normal	2 months	surgery	- VP- shunt right parietal due to triventricular hydrocephalus,<br>- aqueduct stenosis	- control scan two months after surgery	conservative	- clinical control for 4 months without imaging<br>- no symptoms	- asymptomatic<br>- no neuro-psychological exam	scan	no	- left frontal<br><br>- volume not specified<br>- slight mass effect
**44**	**Mehra et al., 2017**	CRP/1	35/M	no	2 months	trauma	- fall from height,<br>- transient loss of consciousness	no	surgery, craniotomy	- surgery without urgence<br>- left occipital and suboccipital craniotomy	- left occipital and suboccipital swelling,<br>- headache<br>- associated hydrocephalus in imaging<br><br>- no neuro-psychological exam	scan	no	- left occipital and suboccipital<br>- volume not specified<br>- slight mass effect with hydrocephalus<br>- Intraoperative: firm gritty collection<br>- no histology<br><br>- special: hydrocephalus and subcutaneous swelling
**45**	**Bayri et al., 2009**	CRP/1	3/F	- not specified	10 months	surgery	- Craniopharyngioma,<br>- bifrontal craniotomy<br>- subduro-peritoneal shunt 2 weeks after surgery due to subdural hygroma	- control- MRI 10 month for follow up:<br>- chronic calcified epidural hematoma with destabilization of the bone flap<br>- tumor recurrence	- surgery due to tumor recurrence and destabilization of bone flap	- surgery without urgence	- asymptomatic,<br>- no neuro-psychological exam	MRI	no	- bifrontal<br>- volume not specified<br>- no mass effect<br>- intraoperative: hematoma encapsulated, firm and thick calcification especially on side of dura<br>- no histology<br>- no complication postoperative

**46**	**Sinha and Borkar, 2008**	CRP/1	14/M	- not specified	8 years	trauma	fall from height	no	- surgery, craniotomy	- surgery without urgence	- mild headaches on- off<br><br>- no neuro-psychological exam	scan	no	- left frontal<br>- volume not specified<br>- mass effect<br>- intraoperative: thick hard calcified wall of hematoma, adherent to dura, good plane between dura and wall of hematoma<br>- no histology<br>- no complication postoperative
**47**	**Kanikomo et al., 2023**	CRP/1	15/M	- not specified<br>- sepsis during stay at ICU	2 month	trauma	traffic accident	Yes,<br>scan: small epidural hematoma right frontal	- surgery, craniotomy	- scan after 3 weeks: beginning resorption<br>- New scan 2 month due to seizure<br><br>- surgery without urgence du to seizure	- hemiparesis<br>- dysarthria<br>- seizure<br>- no neuropsychological exam	scan	no	- right frontal<br>- slight mass effect<br>- no histology<br>- evolution resolution of hemiparesis but seizures stayed
**48**	**Sheyin et al., 2021**	CRP/1	58/M	- not specified	4 years	trauma	traffic accident without loss of consciousness	Yes,<br>X-ray: no fx	- surgery, craniotomy	scan: ossified edh left fronto parietal	- seizures for 4 month<br>- no neuropsychological exam	scan	no	- left frontoparietal<br>- mass effect<br>- no histology<br>- good evolution with resolution of seizures
**49**	**Kumar and Mittal, 2014**	CRP/1	10/F	- routine exam wo pathology	6 yrs	trauma	- fall from tree<br>- following that she developed swelling in right temporal region	no	- surgery, craniotomy	scan: right temporal ossified hematoma with involvement of calvarium	- progressive painless swelling in right temporal region since one year<br>- without any neurological deficient	scan	no	- right temporal region
**50**	**Our case**	CRP/1	22/M	- chronic alcohol abuse<br>- osteogenesis imperfecta	3 years	trauma	- assault-related head trauma with loss of consciousness<br>- history of multiple episodes of head trauma	scan: no intracranial hemorrhage or fracture	- surgery recommended, patient did not show up	- scan<br>- MRI	- the neurological exam was unremarkable<br>- neuropsychological exam: mild-moderate problems	scan: new cranial CT shows a biconvex frontal ossified hematoma	no	- frontal left<br>- left frontoparietal<br>- mass effect<br>- no histology
