# Supplementary material for: Ossification of Cranial Epidural Hematomas: A Systematic Review of Management Strategies and Presentation of an Illustrative Case
Source: Neurotrauma Rep. 2024 Aug 22;5(1):787–99. doi: 10.1089/neur.2024.0065 (PMC11342046; doi:10.1089/neur.2024.0065)
Supplement: Supplementary Appendix S2 [file neur.2024.0065_appendix1.pdf]

Appendix 1

Ovid MEDLINE(R) 1946 to December Week 3 2020
Search was run 30.12.2020

#	searches	results
1	Cranial epidural hematoma.mp. or Hematoma, Epidural, Cranial/	3446
2	Calcification, Physiologic/ or calcification.mp	53165
3	Ossification.mp. or Osteogenesis/	51348
4	2 or 3	100373
5	1 and 4	24

PubMed
Search was run 30.12.2020

#	searches	results
1	(cranial epidural hematoma[MeSH Terms])	3446
2	calcification[MeSH Terms]	58392
3	ossification[Title/Abstract]	19969
4	(calcification[MeSH Terms]) OR ossification ([Title/Abstract])	77290
5	((cranial epidural hematoma[MeSH Terms])) AND ((calcification[MeSH Terms] ossification[Title/abstract]))	27
